# Empowering inclusivity: improving readability of living kidney donation information with ChatGPT

**DOI:** 10.3389/fdgth.2024.1366967

**Published:** 2024-04-10

**Authors:** Oscar A. Garcia Valencia, Charat Thongprayoon, Jing Miao, Supawadee Suppadungsuk, Pajaree Krisanapan, Iasmina M. Craici, Caroline C. Jadlowiec, Shennen A. Mao, Michael A. Mao, Napat Leeaphorn, Pooja Budhiraja, Wisit Cheungpasitporn

**Affiliations:** ^1^ Division of Nephrology and Hypertension, Department of Medicine, Mayo Clinic, Rochester, MN, United States; ^2^ Chakri Naruebodindra Medical Institute, Faculty of Medicine Ramathibodi Hospital, Mahidol University, Samut Prakan, Thailand; ^3^ Division of Nephrology, Department of Internal Medicine, Faculty of Medicine, Thammasat University, Pathum Thani, Thailand; ^4^ Division of Nephrology, Department of Internal Medicine, Thammasat University Hospital, Pathum Thani, Thailand; ^5^ Division of Transplant Surgery, Department of Surgery, Mayo Clinic, Phoenix, AZ, United States; ^6^ Division of Transplant Surgery, Department of Transplant, Mayo Clinic, Jacksonville, FL, United States; ^7^ Division of Nephrology and Hypertension, Department of Medicine, Mayo Clinic, Jacksonville, FL, United States; ^8^ Division of Nephrology and Hypertension, Department of Medicine, Mayo Clinic, Phoenix, AZ, United States

**Keywords:** living kidney donation, disparity, health literacy, inclusivity, ChatGPT

## Abstract

**Background:**

Addressing disparities in living kidney donation requires making information accessible across literacy levels, especially important given that the average American adult reads at an 8th-grade level. This study evaluated the effectiveness of ChatGPT, an advanced AI language model, in simplifying living kidney donation information to an 8th-grade reading level or below.

**Methods:**

We used ChatGPT versions 3.5 and 4.0 to modify 27 questions and answers from Donate Life America, a key resource on living kidney donation. We measured the readability of both original and modified texts using the Flesch-Kincaid formula. A paired *t*-test was conducted to assess changes in readability levels, and a statistical comparison between the two ChatGPT versions was performed.

**Results:**

Originally, the FAQs had an average reading level of 9.6 ± 1.9. Post-modification, ChatGPT 3.5 achieved an average readability level of 7.72 ± 1.85, while ChatGPT 4.0 reached 4.30 ± 1.71, both with a *p*-value <0.001 indicating significant reduction. ChatGPT 3.5 made 59.26% of answers readable below 8th-grade level, whereas ChatGPT 4.0 did so for 96.30% of the texts. The grade level range for modified answers was 3.4–11.3 for ChatGPT 3.5 and 1–8.1 for ChatGPT 4.0.

**Conclusion:**

Both ChatGPT 3.5 and 4.0 effectively lowered the readability grade levels of complex medical information, with ChatGPT 4.0 being more effective. This suggests ChatGPT's potential role in promoting diversity and equity in living kidney donation, indicating scope for further refinement in making medical information more accessible.

## Introduction

Living kidney donation is a vital alternative for individuals diagnosed with end-stage kidney disease (ESKD) who require a kidney transplant (1, 2). It involves a healthy donor, often a family member, giving a kidney to a recipient in need (1, 3). While it has advantages like shorter waiting times and better outcomes, complex medical information can be hard to grasp, especially for those with limited literacy (4–7). Literacy, including reading, writing, and understanding, significantly impacts health literacy, affecting how well individuals comprehend medical options and make informed decisions (8). Addressing this literacy gap is vital for equal access to kidney donation information.

The literacy level in the general population significantly impacts healthcare and patient outcomes (9–14). National Assessment of Adult Literacy (NAAL) reveal that only 12% of adults have proficient health literacy skills, while approximately 22% have basic, and 14% had below basic health literacy (15). The average American adult reads at an eighth-grade level (16, 17), making it challenging to provide medical information suitable for various literacy levels (12). Simplifying living kidney donation information to an eighth-grade level benefits those with limited literacy skills, reducing health disparities and promoting equity in healthcare (4, 18). AI language models like ChatGPT may offer a solution to make complex information more readable (19–21). This study assesses ChatGPT's effectiveness in simplifying living kidney donation information to an eighth-grade level. The need to explore AI language models like ChatGPT for this purpose is evident, as their potential in this context remains understudied.

We collected 27 FAQs related to living kidney donation from Donate Life America (22) and had ChatGPT 3.5 (03/23 Version) (19) simplify both the questions and the answers for those reading at or below an eighth-grade level. We assessed the readability grade level of both the original and modified information using the Flesch-Kincaid formula (23). We then compared the readability before and after the modification and conducted a paired *t*-test to determine any significant differences.

## Method

### Data collection

To gather the necessary data for our study, we accessed a widely accessible FAQs website called Donate Life America (24), which provides information on various aspects of living kidney donation. Donate Life America is a reputable organization dedicated to promoting organ, eye, and tissue donation in the United States. The website serves as a valuable resource for individuals seeking information about living kidney donation. From this website, we collected a total of 27 questions along with their corresponding answers, specifically related to living kidney donation. These questions covered a range of topics, including the eligibility criteria for donors, the evaluation process, the surgical procedure, post-donation care, and potential risks and benefits associated with living kidney donation. The decision to use the Donate Life America website for data collection was based on its accessibility to the public and its reputation as a reliable source of information on living kidney donation. By utilizing content from this website, we aimed to ensure that the information being modified by ChatGPT was representative of the type of information individuals might encounter when seeking information on living kidney donation.

### AI language model usage

In our study, we utilized two versions of OpenAI's ChatGPT (25), GPT-3.5 and the more advanced GPT-4.0, to evaluate their efficacy in modifying living kidney donation information for different literacy levels. ChatGPT, known for its advanced text generation capabilities, was accessed through OpenAI's API. GPT-3.5 was initially used to rephrase information to an eighth-grade reading level. We then employed GPT-4.0, which offers improved language understanding, to adapt the information to even lower literacy levels while maintaining accuracy. The study aimed to assess and compare the effectiveness of both models in simplifying complex medical information for audiences with lower literacy, exploring how advancements in AI can enhance readability and accessibility.

### Readability assessment

The process involved providing the original text of the answers as prompts to ChatGPT and receiving modified versions of the text in response. ChatGPT utilizes its understanding of language and context to generate modified versions that are expected to be more accessible to individuals with lower literacy skills. These modifications aim to reduce complexity, simplify sentence structures, and clarify any ambiguous or technical terms present in the original answers.

To assess the readability of the original and modified living kidney donation information, we employed the widely-used Flesch-Kincaid formula (23, 26). The Flesch-Kincaid formula is a readability formula that measures the complexity of a text and assigns it a grade level. It considers factors such as sentence length and average number of syllables per word to determine the reading level required to comprehend the text effectively (27, 28).

The formula calculates the grade level based on the following equation (23):

GradeLevel=0.39×(TotalWords/TotalSentences)+11.8×(TotalSyllables/TotalWords)−15.59

To apply the Flesch-Kincaid formula, we first needed to count the total number of words, sentences, and syllables in each answer. This was done by utilizing software tools specifically designed for text analysis or by manual counting. The number of syllables in a word can be determined by counting the number of vowels sounds in the word.

Once we obtained the necessary counts, we substituted them into the Flesch-Kincaid formula to calculate the grade level of each answer. This grade level represents the minimum level of education required to understand the text. Lower grade levels indicate easier readability, while higher grade levels indicate more complex language.

By applying the Flesch-Kincaid formula to both the original and modified answers, we were able to compare the grade levels before and after ChatGPT's modifications. This comparison provided insights into the effectiveness of ChatGPT in reducing the complexity and improving the readability of the living kidney donation information.

### Accuracy and fidelity verification

Upon completing the initial modifications by ChatGPT, each modified text underwent a rigorous review process.

• Medical accuracy: Ensuring that the modified content accurately reflected current medical knowledge and practices related to living kidney donation.
• Fidelity to the original message: Confirming that key information, advice, and implications remained unchanged from the original texts.
• Clarity and comprehensibility: Assessing whether the modifications indeed made the information more accessible to individuals with lower literacy levels, without sacrificing the depth of information.

A second attempt at modifying each of the 27 questions and their answers was conducted using ChatGPT. To ensure the independence of this attempt and to minimize potential biases arising from the AI's memory of prior interactions, we initiated this process in a new chat session for each question and answer. This procedural adjustment allowed us to assess the reproducibility of ChatGPT's text modifications and to examine the variability in readability levels across different attempts.

### Statistical analysis

Our analysis compared the readability grades pre- and post-modification by ChatGPT 3.5 and 4.0. We employed a paired *t*-test to determine the significance of readability enhancements. Additionally, a statistical comparison between the two ChatGPT versions was conducted, focusing on the mean difference (MD) and standard deviation (SD). This comparative analysis aimed to evaluate the effectiveness of each version in simplifying medical information to the target literacy level.

## Results

### Original readability assessment

Upon analysis of the 27 questions and answers related to living kidney donation using the Flesch-Kincaid formula, the original texts exhibited an average readability grade level of 9.6 ± 1.9. This level was indicative of the texts' complexity, surpassing the average American adult's reading ability (Table 1).

**Table 1:** Comparison of original and modified text readability grade levels for 27 questions related to living kidney donation.

Question Number	Question	Readability of original answer	Readability level after ChatGPT 3.5	Readability level after ChatGPT 4.0	Difference (Original—ChatGPT 3.5)	Difference (Original—ChatGPT 4.0)
1	What is living donation?	9.1	6.5	5.3	2.6	3.8
2	Why is living donation important?	10.7	8.4	5.3	2.3	5.4
3	Does the living donor need to know the person…	6.1	5.5	3.4	0.6	2.7
4	What is involved in the evaluation to be a living donor?	9.2	8.4	4.3	0.8	4.9
5	What is paired kidney donation and living donation chains?	9.5	6.5	2.7	3	6.8
6	Can living kidney donors live a healthy life with a single kidney?	8.5	6.9	5.2	1.6	3.3
7	Does living organ donation shorten the donor's life expectancy?	8.5	6.6	3.7	1.9	4.8
8	Are living kidney donors more likely to get kidney disease?	11.5	10.4	8.1	1.1	3.4
9	Do living donors have to take medications for the rest of their lives?	11.9	7.6	6.6	4.3	5.3
10	Will a living donor be in the hospital for an extended period of time after surgery?	7.6	8.5	5	−0.9	2.6
11	Does a living donor have to follow a new diet plan following donation?	8.8	8	3.2	0.8	5.6
12	Can a living donor consume alcohol following donation?	11.2	8.5	6	2.7	5.2
13	Should a living donor avoid pregnancy after donation?	10.7	8	5	2.7	5.7
14	Will a living donor's sex life be negatively affected by donation?	7.3	3.4	4.1	3.9	3.2
15	Who pays for the medical expenses related to living donation?	10.8	10.3	6.4	0.5	4.4
16	Will the living donor have any out of pocket expenses?	11	11.3	6	−0.3	5
17	Will a living donor have trouble getting health insurance or life insurance after they donate?	8.2	6.7	2.8	1.5	5.4
18	How risky is living donor surgery?	10	7.2	5	2.8	5
19	Can adults over age 50 donate?	6.3	5.1	1	1.2	5.3
20	Can members of the LGBTQ+ community be living donors?	11.2	6.2	2.3	5	8.9
21	Can people living with HIV be living donors?	12	7.6	1.3	4.4	10.7
22	Are people with tattoos able to be living donors?	11.9	7.8	2.3	4.1	9.6
23	What happens if a living donor changes their mind?	7.5	5.5	3.4	2	4.1
24	Do living donors ever share their experiences and what are some resources?	9.3	8.4	3.2	0.9	6.1
25	How would a potential living donor start the process if they want to donate to a specific person?	11.5	11	5.8	0.5	5.7
26	What if the intended living kidney donor and intended recipient are not a match?	11.6	9.7	5.3	1.9	6.3
27	What is the first step for a potential living donor who does not have a specific recipient in mind?	6.5	8.5	3.3	−2	3.2

**Figure 1:**
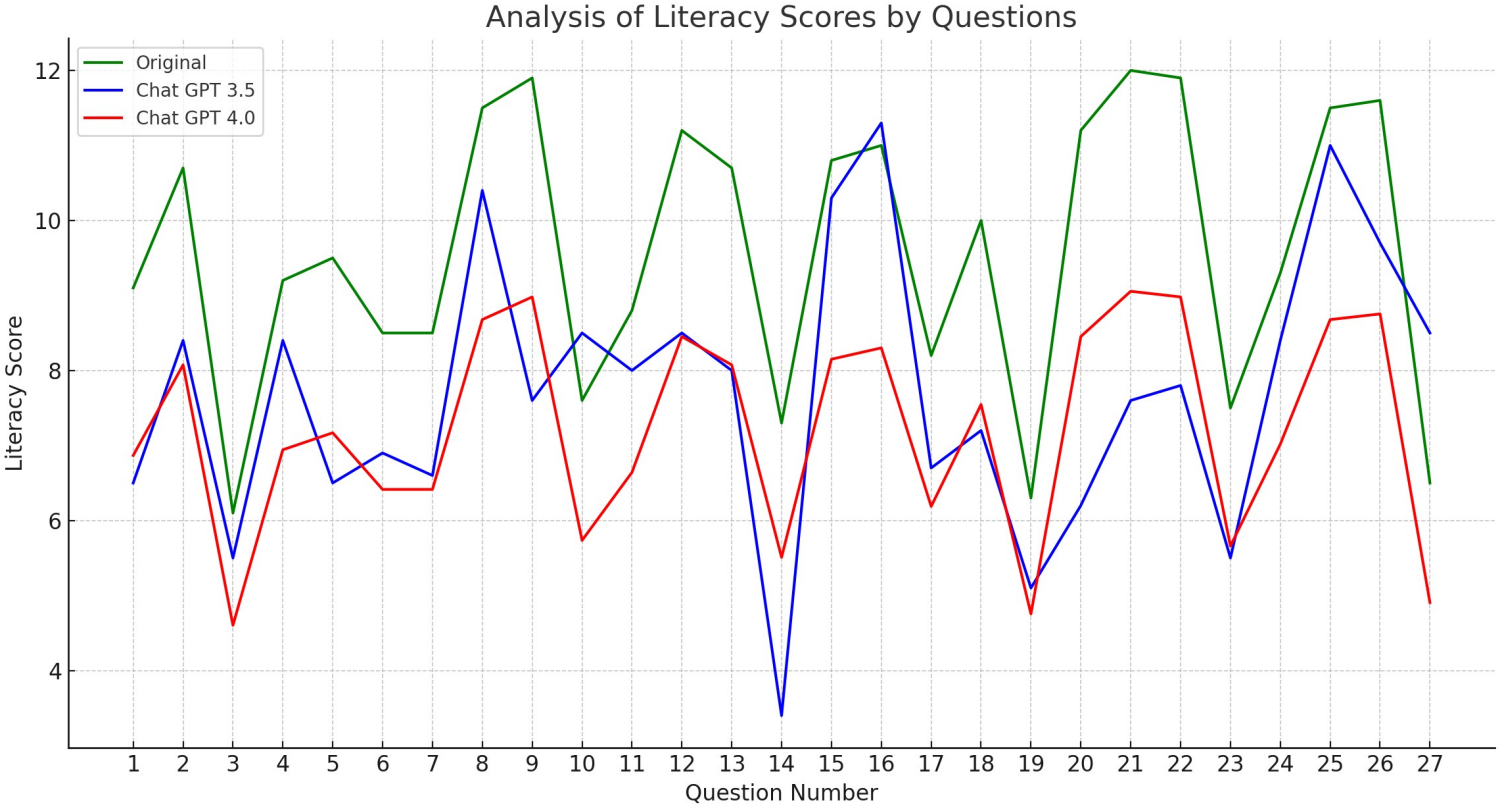
Analysis of literacy scores by questions. Three lines represent the scores from the original text, and the modified versions by Chat GPT 3.5 and Chat GPT 4.0, respectively. The y-axis indicates the literacy score, which presumably reflects the readability level or grade level of the text, with the x-axis enumerating the individual questions.

When modified by ChatGPT 3.5, the average readability grade level of these FAQs decreased to 7.72 ± 1.85, showcasing a significant readability enhancement (*p* < 0.001). However, ChatGPT 3.5 managed to adjust the content to an eighth-grade reading level or below in 16 of the 27 cases (59.26%), Figure 1. In contrast, the utilization of ChatGPT 4.0 led to a more pronounced reduction in complexity, with the average readability level dropping to 4.30 ± 1.71. Notably, ChatGPT 4.0 successfully reduced the readability level to the eighth grade or below in 26 of the 27 instances (96.30%), demonstrating its superior capability in simplifying complex medical information.

The range of grade levels for the modified answers was notably different between the two versions of ChatGPT. For ChatGPT 3.5, this range spanned from 3.4 to 11.3, indicating variability in its effectiveness. In contrast, ChatGPT 4.0 consistently produced answers within a more accessible range of 1–8.1. The mean difference (MD) in grade levels between the original and modified answers was 3.43 (SD = 1.62, *p*-value <0.001), highlighting the substantial impact of these AI models in enhancing readability.

The most substantial enhancements in readability, as reflected by the contrast between the initial levels and those after modification by ChatGPT versions 3.5 and 4.0, were seen in five key questions (Table 1). Question 21, regarding the eligibility of individuals living with HIV for organ donation, saw the most significant improvement, with the readability level plunging from 12 to 1.3, a difference of 10.7. Close behind, Question 20's discussion on the possibility of LGBTQ+ community members becoming donors improved from a readability level of 11.2–2.3, marking an 8.9 point enhancement. Similarly, Question 22's query about the eligibility of tattooed individuals as donors improved by 9.6 points, Question 24's exploration of donor experiences and resources by 6.1 points, and Question 5's explanation of paired kidney donation and donation chains by 6.8 points. These significant reductions in readability scores indicate a notable increase in the accessibility of information, making it potentially easier to comprehend for a broader audience.

In the assessment of readability changes following modifications by ChatGPT versions, certain questions displayed negligible enhancement or even regression. For instance, Question 10, concerning the duration of hospital stay post-donation, regressed in readability, increasing from a grade level of 7.6–8.5. Similarly, Question 16 saw a slight increase in complexity, with the readability level marginally rising from 11 to 11.3. Question 27 also experienced a decline in readability, as the grade level went up from 6.5 to 8.5. While Questions 15 and 25 showed only minimal improvements, with the readability level slightly decreasing from 10.8 to 10.3 and from 11.5 to 11, respectively. These instances indicate either a deterioration in the simplicity of the information or a minor readability enhancement, which might not significantly aid in understanding for the intended audience.

### Second attempt results

The second attempt at modifying the texts with both ChatGPT 3.5 and ChatGPT 4.0 reaffirmed the initial findings, with no significant differences observed in the readability levels or the content's fidelity. This consistency underscores the reliability of ChatGPT in simplifying complex medical texts. Specifically, the average readability grade level for the FAQs remained substantially reduced, mirroring the results from the first attempt:

• With ChatGPT 3.5, the readability level averaged at 7.75 ± 1.82, closely aligning with the initial average of 7.72 ± 1.85.
• ChatGPT 4.0 demonstrated a remarkable consistency as well, with the second attempt yielding an average readability level of 4.32 ± 1.69, which is virtually identical to the first attempt's average of 4.30 ± 1.71.

### Accuracy in simplifying living kidney donation information

Both versions of ChatGPT achieved 100% accuracy in simplifying the texts while perfectly preserving the medical integrity and fidelity of the information. This high level of accuracy represents a significant milestone in using AI to make complex medical information more accessible without compromising content quality (Supplementary Data Sheet S1).

For instance, when simplifying the concept of “living donation”, ChatGPT 4.0 skillfully condensed the original explanation into a clear and easily understandable version. It transformed the complex description of living donation—a process where a living individual donates an organ or part of an organ to someone in need, with specifics about kidney and liver donation—into a concise explanation. ChatGPT 4.0 clarified that living donation means a person who is alive gives an organ or a part of an organ to someone who needs a new one, emphasizing the body's ability to function well even after such a donation.

Likewise, when addressing “Why is living donation important?”, ChatGPT 4.0 effectively distilled the multifaceted reasons into a digestible format. It highlighted the significance of providing another option for transplant candidates and alleviating the long wait times associated with receiving organs from deceased donors. The simplification successfully conveyed the urgency of living donation in meeting the demand for life-saving transplants and its benefits for the recipient's health and the quality of the donated organ.

### Fidelity to the original message

The examination of the simplified explanations provided by ChatGPT 4.0 demonstrates a high level of fidelity to the original content. In the example discussing paired kidney donation and living donation chains (Supplementary Data Sheet S1), ChatGPT 4.0 accurately conveys the key concepts presented in the original text. The simplified explanation maintains the essential information about the challenges of finding a compatible donor and the solution offered by paired donation, where multiple donor-recipient pairs are matched to ensure compatibility. The model effectively preserves the core message and intent of the original text while presenting the information in a more accessible manner.

Similarly, when addressing concerns about the impact of living donation on pregnancy and sex life, ChatGPT 4.0 maintains fidelity to the original message. The simplified responses accurately reflect the advice provided in the original text, emphasizing the importance of waiting for medical clearance before attempting to conceive after donation and reassuring potential donors that donation does not necessarily negatively impact their sex life. The model successfully distills the essential information and guidance from the original text, ensuring that the simplified explanations remain faithful to the intended message.

### Clarity and comprehensibility

The simplified explanations provided by ChatGPT 4.0 demonstrate a significant improvement in clarity and comprehensibility compared to the original text. In the example discussing paired kidney donation and living donation chains (Supplementary Data Sheet S1), ChatGPT 4.0 uses relatable language and an example scenario to help the reader understand the concept. By avoiding complex medical terminology and presenting the information in a narrative format, the model makes the explanation more accessible to individuals with lower literacy levels. The use of phrases like “kidney swap” and the step-by-step description of how paired donation works enhance the clarity of the explanation, making it easier for readers to grasp the key points.

Similarly, when addressing concerns about pregnancy and sex life after donation, ChatGPT 4.0 provides clear and concise responses that are easy to understand. The simplified answers directly address the questions at hand, using straightforward language that is accessible to a wide audience. By focusing on the essential information and avoiding unnecessary details, the model enhances the comprehensibility of the guidance provided. The use of short, direct sentences further improves clarity, making it easier for readers to understand the key points and recommendations.

## Discussion

The use of advanced language models such as ChatGPT 3.5 and 4.0 in making complex medical information more accessible represents a significant stride in the pursuit of health literacy. Our findings reveal ChatGPT's effectiveness in decreasing the readability grade level of FAQs regarding living kidney donation, thereby making strides towards broader understanding and paving the way for improved equity in health communication. Despite the progress, the goal of consistently achieving an eighth-grade reading level across all materials has not been fully realized, highlighting an ongoing challenge in the quest for equitable health literacy.

The notable performance of ChatGPT 4.0, in particular, suggests that further developments in AI can lead to even more effective simplification of medical language. The demonstrated correlation between the complexity of the original texts and the degree of readability enhancements provided by these AI models suggests a targeted application of such technology could be exceptionally beneficial in refining materials that are typically dense and challenging for non-specialist audiences.

The persistent literacy gaps in healthcare represent a barrier to equitable health information access (7, 12, 14, 29). Despite the vast amount of medical knowledge available, there is a disparity in the ability of individuals to understand and act upon health-related information (4, 9, 29, 30). This gap is not merely a reflection of individual educational achievements but is also a systemic issue that affects public health outcomes (14, 31). In the context of living kidney donation, the stakes are particularly high, as the decision to donate or receive a kidney involves navigating complex medical procedures and understanding the long-term implications for both donor and recipient (1, 5, 18, 32). As such, ensuring that information regarding living kidney donation is accessible to people of all educational backgrounds is not just a matter of convenience; it is a matter of ethical necessity and health equity (33).

Policies aimed at closing the literacy gaps in healthcare must take into account the diversity of patients' educational backgrounds (29, 34–36). A comprehensive strategy should include the simplification of medical texts, the use of plain language in patient education materials, and the training of healthcare providers to communicate effectively with patients who may have limited health literacy (35, 36). Furthermore, policy initiatives must also foster the development and deployment of advanced technologies, such as AI-driven tools like ChatGPT, which can tailor complex medical content to the comprehension skills of the general population. By embedding these strategies into healthcare policy (34), institutions can better ensure that all individuals, regardless of literacy level, have the information they need to make informed decisions about living kidney donation.

Living kidney donation holds profound importance for public health, as it often represents a life-saving intervention for individuals with end-stage renal disease. The decision to donate a kidney is a significant one, with far-reaching consequences for the health and well-being of both the donor and the recipient. Therefore, it is critical that every person considering living kidney donation has access to clear and comprehensible information (1, 4, 32). This is not just a matter of providing education; it is about upholding the rights of individuals to make autonomous decisions about their bodies and health. Ensuring that everyone, irrespective of their educational attainment, can fully understand the implications of living kidney donation is a step towards upholding the principles of justice and equality in healthcare.

To fully bridge the literacy gap, it is essential to iteratively improve AI models like ChatGPT, focusing on language nuances and information accessibility. Future developments should aim for language simplification, semantic clarity, and cultural relevance to make health information inclusive. Research should expand to include various medical sources and assess the impact on patient comprehension and outcomes. This study underscores the potential of AI in making medical content more universally understandable at an eighth-grade level, aiding informed decision-making and supporting healthcare diversity and equity. Ongoing refinement of these AI tools is crucial, enhancing their ability to deliver engaging, culturally sensitive medical information. Ensuring equitable access to information for all literacy levels is key to informed consent and patient empowerment, and the development of these technologies should prioritize inclusivity in the evolving health information landscape.

The study highlights the significant potential of ChatGPT in making medical information more accessible. However, it has limitations. The data was sourced only from Donate Life America's FAQs, limiting the generalizability of our findings across diverse medical texts. Additionally, while our study demonstrates the effectiveness of ChatGPT in improving the readability of medical information on living kidney donation using the Flesch-Kincaid Index, it is important to acknowledge the limitations of this readability measure. The Flesch-Kincaid Index, although widely used, it may not fully capture the intricacies of readability, such as the use of jargon, the complexity of ideas, or the coherence of the text (37). Furthermore, it is crucial to include a nuanced discussion on the appropriateness and limitations of using the eighth-grade readability level as a universal target. Acknowledging the diversity of literacy skills within the population, future studies should explore more inclusive strategies. These could include developing tiered content that caters to varying literacy demands and integrating visual aids to support comprehension for those at the lower end of the literacy spectrum. To address these limitations, future studies should consider incorporating additional readability indices, such as the Gunning Fog Index and the SMOG Index (37–40). Furthermore, given the technologies available, an adaptive preparation of the information would be a promising option. Additionally, future studies could explore the relationship between different readability indices and the actual comprehension and retention of information by the target audience. This could involve conducting user studies with participants of varying literacy levels to assess the effectiveness of the simplified texts in promoting understanding and knowledge acquisition. Such research would provide valuable insights into the practical implications of readability enhancements and help refine the text simplification strategies employed by AI tools like ChatGPT.

In conclusion, this study demonstrates that ChatGPT's potential in bridging the literacy gap in understanding the living kidney donation information. By tailoring the content to resonate with an eighth-grade comprehensive level, ChatGPT broadens the reach and clarity of information for those with different literacy levels. This endeavor aligns with the overarching aim of amplifying diversity and equality in living kidney donation by ensuring inclusivity in information dissemination. Nonetheless, further research and AI development are necessary to finetune tools like ChatGPT, and enhance their performance in tailoring medical information for diverse literacy individuals.

## Data Availability

The original contributions presented in the study are included in the article/**Supplementary Material**, further inquiries can be directed to the corresponding author.
