# Supplementary material for: Empowering inclusivity: improving readability of living kidney donation information with ChatGPT
Source: Front Digit Health. 2024 Apr 10;6:1366967. doi: 10.3389/fdgth.2024.1366967 (PMC11039889; doi:10.3389/fdgth.2024.1366967)
Supplement: Supplementary file 1 [file Datasheet1.pdf]

1. Please modify the text to make it easier to understand for someone who reads at or below an 8th grade level: "What is living donation? Living donation is when a living person donates an organ or part of an organ to a person in need of a transplant. Living kidney donation is possible because we can live a healthy life with one functioning kidney. Living liver donation is possible because the liver consists of two lobes, one of which can be donated to someone in need. Both lobes will regenerate to normal size and function generally within 6-12 weeks."

ChatGPT

ChatGPT

What does living donation mean? Living donation is when someone who's alive gives an organ or a part of an organ to help someone who needs a transplant. For example, with kidneys, we have two, but we can live well with just one. With the liver, it has two parts, and one of them can be given to someone who needs it. The part that's given will grow back to its normal size and work as it should in about 6 to 12 weeks.

CH

You

2. Please modify the text to make it easier to understand for someone who reads at or below an 8th grade level: "Why is living donation important? Living donation offers another choice for transplant candidates, and reduces the number of people on the national transplant waiting list (from deceased donors). Even better, kidney and liver patients who are able to receive a living donor transplant can receive a good quality organ much sooner, often in less than a year. The limited number of deceased donor organs cannot meet the growing need for lifesaving organ transplants. Living donation is one way we can give more people access to the lifesaving transplant they need. Other benefits of living donation include: The recipient can be transplanted while in better health and better able to tolerate the surgery. Living donor organs are often better quality and on average last longer than deceased donor organs. Kidney disease patients can avoid years of dialysis. The surgery can be scheduled at a time convenient for both the donor and the recipient. Many living donors refer to the experience as one of their most rewarding achievements."

ChatGPT

ChatGPT

Why is living donation important? It gives another option for people waiting for a transplant and helps reduce the number of people waiting for organs from those who have passed away. Also, if someone gets a kidney or liver from a living donor, they can often get a good organ much faster, usually within a year. Since there aren't enough organs from people who have passed away to meet the demand, living donation helps more people get the life-saving transplants they need.

There are other benefits too:

- The person getting the transplant is usually healthier and can handle the surgery better.
- Organs from living donors are often better and last longer than those from people who have passed away.
- People with kidney problems can avoid spending years on dialysis.
- The surgery can be planned at a convenient time for both the donor and the recipient.
- Many donors say it's one of the best things they've ever done.

CH

You

3. Please modify the text to make it easier to understand for someone who reads at or below an 8th grade level: "Does the living donor need to know the person to whom they donate an organ? No. A person may donate to a specific person they know or anonymously to someone in need. This would be a non-directed donation."

ChatGPT

ChatGPT

Does the person giving their organ need to know the person receiving it? No. You can give to someone you know or to someone you don't know. This is called a non-directed donation.

CH

You

4. Please modify the text to make it easier to understand for someone who reads at or below an 8th grade level: "What is involved in the evaluation to be a living donor? The evaluation is designed to protect both the donor and the recipient. It ensures that the donor is healthy enough for the surgery and is making an informed decision. A potential living donor undergoes both physical and psychosocial examinations. Testing can vary depending on the organ and donor’s age. In addition, routine health screens will need to be up to date before donation can occur. Learn more about living donation health screens from the American Society of Transplantation (AST)."

ChatGPT

ChatGPT

What happens when you want to donate an organ while you're alive? The evaluation process is there to make sure you're healthy and making the right choice. It includes physical and mental check-ups to ensure you're fit for surgery and understand what you're doing. The tests might be different depending on your age and the organ you're donating. Plus, you need to have all your regular health checks done before you can donate. You can find out more about these health checks from the American Society of Transplantation (AST).

CH

You

5. Please modify the text to make it easier to understand for someone who reads at or below an 8th grade level: "What is paired kidney donation and living donation chains? Sometimes a transplant candidate has someone who wants to donate a kidney to them, but tests reveal that the kidney would not be a good medical match. Paired donation, also called paired kidney exchange, gives that transplant candidate another option. In paired donation, two or more pairs of living kidney donors and the individuals they want to donate to are swapped to form a compatible medical match. For more detailed information, check with your transplant hospital or learn more about paired kidney donation from UNOS.org"

ChatGPT

ChatGPT

What's paired kidney donation and living donation chains? Well, sometimes a person needs a kidney, and they have someone willing to donate one to them. But tests show that the donated kidney wouldn't work well for them. That's where paired donation comes in. It's like a kidney swap. Two or more pairs of people who want to donate and receive kidneys exchange donors so that everyone

ends up with a good match. For more info, ask your transplant hospital or check out UNOS.org to learn about paired kidney donation.

CH

You

6. Please modify the text to make it easier to understand for someone who reads at or below an 8th grade level: "Can living kidney donors live a healthy life with a single kidney? Studies suggest that people can live a normal, healthy life with one kidney. Living kidney donors are carefully screened to ensure they are healthy before living donation can take place. They are encouraged to have regular checkups and follow a healthy lifestyle."

ChatGPT

ChatGPT

Can people who donate one kidney live a healthy life? Research shows that yes, they can. Before donating, they go through tests to make sure they're healthy. After donation, they're advised to have regular checkups and stay healthy.

CH

You

7. Please modify the text to make it easier to understand for someone who reads at or below an 8th grade level: "Does living organ donation shorten the donor's life expectancy? No, there is no evidence that living donation shortens a lifespan."

ChatGPT

ChatGPT

Does donating an organ while you're alive make your life shorter? No, there's no proof that it does.

CH

You

8. Please modify the text to make it easier to understand for someone who reads at or below an 8th grade level: "Are living kidney donors more likely to get kidney disease? Living kidney donors are counseled about their individual risks associated with donation. The transplant team educates donors about the expected post-donation kidney function. In general, the risk of kidney disease does not exceed that of the general population with the same demographic profile."

ChatGPT

ChatGPT

Do people who donate a kidney while they're alive have a higher chance of getting kidney disease? Donors talk with the transplant team to understand their own risks. They're told what to expect after donating. Usually, their risk of kidney disease isn't higher than other people with similar backgrounds.

CH

You

9. Please modify the text to make it easier to understand for someone who reads at or below an 8th grade level: "Do living donors have to take medications for the rest of their lives? Generally, prescriptions for pain and stool softeners will be necessary in the immediate post-operative period only."

ChatGPT

ChatGPT

Do donors have to take medicines forever? Usually, they only need painkillers and stool softeners right after the surgery, not for the rest of their lives.

CH

You

10. Please modify the text to make it easier to understand for someone who reads at or below an 8th grade level: "Will a living donor be in the hospital for an extended period of time after surgery? A kidney donor will be hospitalized on average for 1 to 2 nights. A liver donor will be hospitalized on average for 4 to 7 nights. Liver donors may spend at least 1 night in the ICU for monitoring. A donor will have some follow-up appointments after hospital discharge."

ChatGPT

ChatGPT

Will donors stay in the hospital for a long time after surgery? Usually, a kidney donor stays in the hospital for 1 to 2 nights. A liver donor might stay for 4 to 7 nights, and they might spend at least 1 night in the ICU for extra care. After leaving the hospital, donors will have some follow-up appointments.

CH

You

11. Please modify the text to make it easier to understand for someone who reads at or below an 8th grade level: "Does a living donor have to follow a new diet plan following donation? A donor should eat a healthy, well-balanced diet, but there are no dietary restrictions."

ChatGPT

ChatGPT

After donating, does the donor need to follow a special diet? It's important for donors to eat healthy and balanced meals, but they don't have to follow any specific diet plan or avoid certain foods.

CH

You

12. Please modify the text to make it easier to understand for someone who reads at or below an 8th grade level: "Can a living donor consume alcohol following donation? Excessive alcohol use is always dangerous. For liver donors, alcohol abstinence should occur for one month prior to surgery and for three months following donation. No specific restrictions for kidney donors."

ChatGPT

ChatGPT

After donating, can donors drink alcohol? Drinking too much alcohol is never safe. But for liver donors, it's important to avoid alcohol for one month before surgery and for three months after donation. There aren't any specific restrictions for kidney donors.

CH

You

13. Please modify the text to make it easier to understand for someone who reads at or below an 8th grade level: "Should a living donor avoid pregnancy after donation? A donor should wait to become pregnant until medically cleared after donation."

ChatGPT

ChatGPT

Should someone who donates an organ avoid getting pregnant afterward? It's best for donors to wait until they get the OK from their doctor before trying to get pregnant.

CH

You

14. Please modify the text to make it easier to understand for someone who reads at or below an 8th grade level: "Will a living donor's sex life be negatively affected by donation? Donors may engage in sexual activity when they feel well enough to do so."

ChatGPT

ChatGPT

Will donating an organ affect a donor's sex life? Donors can resume sexual activity when they feel up to it.

CH

You

15. Please modify the text to make it easier to understand for someone who reads at or below an 8th grade level: "Who pays for the medical expenses related to living donation? The costs for the medical testing, surgery and post-operative care are covered by the transplant hospital or recipient's health insurance. However, the living donor is responsible for the cost of routine health screens and treatment of any medical issues discovered during the evaluation. The cost of treatment of future issues that result from the donation, may be covered. Medical costs that are not covered include post-surgical routine health maintenance. Employed living donors should learn about their company's paid sick leave, disability and the Family and Medical Leave Act (FMLA) policies if applicable. A transplant financial coordinator is available at each transplant hospital to answer questions."

ChatGPT

ChatGPT

Who pays for the medical stuff when someone donates an organ? The transplant hospital or the recipient's health insurance covers the costs of tests, surgery, and care after the operation. But the donor has to pay for regular health check-ups and any treatment needed because of issues found during the tests. Sometimes, the costs of future problems caused by the donation are covered. But things like normal check-ups after surgery aren't covered. If you have a job, it's good to know about your sick leave and disability policies. If you're eligible, you can also check out the Family and Medical Leave Act (FMLA). Each transplant hospital has a financial coordinator who can help answer questions.

CH

You

16. Please modify the text to make it easier to understand for someone who reads at or below an 8th grade level: "Will the living donor have any out of pocket expenses? Yes. It varies case by case and could include lost wages, travel expenses, childcare costs, etc. The National Organ Transplant Act allows for organ recipients to pay their living donors' travel and housing in connection with the donation. The transplant hospital financial coordinator will check with the insurance company of the intended recipient as the policy may provide a travel benefit. There are organizations that provide financial resources to living donors for uncovered costs depending on need. Transplant hospitals can

provide additional information about options. National Living Donor Assistance Center (NLDAC) Note that eligibility for financial assistance from NLDAC is based on the recipient’s household income which may not be more than 300% of the current Federal Poverty Guidelines."

ChatGPT

ChatGPT

Will the donor have to pay for anything? Yes. It depends on the situation and could include things like missed work, travel expenses, or childcare. The National Organ Transplant Act allows organ recipients to cover their living donor's travel and housing costs related to the donation. The transplant hospital's financial coordinator can check if the recipient's insurance includes a travel benefit. There are also organizations that offer financial help to donors for costs not covered by insurance, depending on their needs. The transplant hospital can give more info on these options. One organization to check out is the National Living Donor Assistance Center (NLDAC). Keep in mind that to get financial help from NLDAC, the recipient's household income must not be more than 300% of the current Federal Poverty Guidelines.

CH

You

17. Please modify the text to make it easier to understand for someone who reads at or below an 8th grade level: "Will a living donor have trouble getting health insurance or life insurance after they donate? Being a living donor may affect the ability to get health or life insurance. The transplant team can provide details."

ChatGPT

ChatGPT

Will donating an organ make it hard to get health or life insurance later on? It might. The transplant team can give more information about this.

CH

You

18. Please modify the text to make it easier to understand for someone who reads at or below an 8th grade level: "How risky is living donor surgery? The surgery to donate a kidney is generally safe, with a 3 in 10,000 risk of death. The risks for living liver donation surgery are slightly higher, dependent on the amount of liver donated. More information about living donation risks is available on the United Network for Organ Sharing (UNOS) website, TransplantLiving.org. As with any surgery, living-liver donation can include complications such as bleeding, infection, bile duct problems, and blood clots, etc. Complications occur in about 16% of cases. Living donors are carefully evaluated prior to a living-donor liver transplant to ensure that risk is minimized. The estimated mortality is about 0.5% (5/1000), depending on which lobe (part) of the liver is donated."

ChatGPT

ChatGPT

How risky is it to have surgery to donate an organ while you're alive? Donating a kidney is usually safe, with a very low risk of death, about 3 in 10,000. Donating part of your liver is a bit riskier, depending on how much you donate. You can find more info about the risks of living donation on the United Network for Organ Sharing (UNOS) website or TransplantLiving.org. Like any surgery, there can be problems such as bleeding, infection, or blood clots. Complications happen in about

16% of cases. Before a living-liver donation, donors are carefully checked to lower the risks. The chance of death is estimated to be around 0.5%, depending on which part of the liver is donated.

CH

You

19. Please modify the text to make it easier to understand for someone who reads at or below an 8th grade level: "Can adults over age 50 donate? Yes, donors must be healthy and can donate primarily on a basis of health. Some transplant hospitals may have age restrictions."

ChatGPT

ChatGPT

Can people over 50 donate? Yes, as long as they're healthy. Some hospitals might have rules about age, though.

CH

You

20. Please modify the text to make it easier to understand for someone who reads at or below an 8th grade level: "Can members of the LGBTQ+ community be living donors? There are no restrictions based on a potential donor's LGBTQ+ identification. All potential living donors undergo a thorough medical evaluation to determine if donation is safe."

ChatGPT

ChatGPT

Can LGBTQ+ people donate organs while they're alive? Yes, there aren't any rules against it because of someone's LGBTQ+ identity. Everyone who wants to donate gets checked by doctors to make sure it's safe for them to do so.

CH

You

21. Please modify the text to make it easier to understand for someone who reads at or below an 8th grade level: "Can people living with HIV be living donors? The HIV Organ Policy Equity Act (HOPE) Act provides donation and transplantation opportunities for people living with HIV with minimal additional testing at specific centers. Refer to the OPTN HOPE (HIV Organ Policy Equity) Act page for more information."

ChatGPT

ChatGPT

Can people with HIV donate organs while they're alive? Yes, the HIV Organ Policy Equity Act (HOPE Act) allows it at certain centers with just a bit of extra testing. You can find more info on the OPTN HOPE (HIV Organ Policy Equity) Act page.

CH

You

22. Please modify the text to make it easier to understand for someone who reads at or below an 8th grade level: "Are people with tattoos able to be living donors? All potential living donors undergo a thorough medical evaluation to determine if donation is safe for both the donor and recipient."

ChatGPT

ChatGPT

Can people with tattoos donate organs while they're alive? Everyone who wants to donate gets checked by doctors to make sure it's safe for both them and the person receiving the organ.

CH

You

23. Please modify the text to make it easier to understand for someone who reads at or below an 8th grade level: "What happens if a living donor changes their mind? Being a living donor is completely voluntary. The donor can delay or stop the process at any time. Their reasons for choosing to not donate will remain confidential and will not be shared with the recipient or anyone else."

ChatGPT

ChatGPT

What if someone who wanted to donate changes their mind? Donating is a choice, and the donor can change their mind anytime. They don't have to go through with it if they don't want to. The reasons for their decision will be kept private and won't be told to the person getting the organ or anyone else.

CH

You

24. Please modify the text to make it easier to understand for someone who reads at or below an 8th grade level: "Do living donors ever share their experiences and what are some resources? Yes, 97% of living donors say they would donate again and are excited to share their experiences. Donate Life WELD (We Encourage Living Donation), a program of Donate Life America, provides a forum for living donors to share their stories with members of their community. To learn more about DL/WELD, contact DL/WELD coordinator. View stories from living donors and recipients."

ChatGPT

ChatGPT

Do people who donate organs while they're alive talk about their experiences? Yes, almost all of them say they'd do it again and are happy to share their stories. Donate Life WELD (We Encourage Living Donation), a program by Donate Life America, gives donors a place to share their stories with others in their community. To find out more about DL/WELD, you can contact the DL/WELD coordinator. You can also read stories from donors and recipients.

CH

You

25. Please modify the text to make it easier to understand for someone who reads at or below an 8th grade level: "How would a potential living donor start the process if they want to donate to a specific person? Contacting the transplant hospital of the person in need of the transplant is the first step. Many hospitals have a form on their website potential donors can submit to begin the process or they can call the transplant hospital and ask to speak with the living donor coordinator for the organ they wish to donate."

ChatGPT

ChatGPT

If someone wants to donate to a specific person, how do they start? The first step is to get in touch with the transplant hospital where the person who needs the transplant is being treated. Many hospitals have a form on their website that potential donors can fill out to start the process. Or, they

can call the transplant hospital and ask to speak with the living donor coordinator for the organ they want to donate.

CH

You

26. Please modify the text to make it easier to understand for someone who reads at or below an 8th grade level: "What if the intended living kidney donor and intended recipient are not a match? Begin by talking to the transplant hospital where the intended recipient is listed to see if they participate in paired donation. The OPTN Kidney Paired Donation Program facilitates paired donation between transplant hospitals."

ChatGPT

ChatGPT

What if the person who wants to donate a kidney and the person who needs it aren't a match? Start by talking to the hospital where the person who needs the kidney is listed. See if they do something called paired donation. The OPTN Kidney Paired Donation Program helps hospitals do this kind of donation swapping between donors and recipients.

CH

You

27. Please modify the text to make it easier to understand for someone who reads at or below an 8th grade level: "What is the first step for a potential living donor who does not have a specific recipient in mind? Please refer to the Organ Procurement and Transplantation Network (OPTN) member list of all U.S. transplant hospitals. You may also want to consider being part of a living kidney donor paired exchange or chain."

ChatGPT

ChatGPT

What should someone do if they want to donate but don't know who needs the organ? First, check the list of all U.S. transplant hospitals on the Organ Procurement and Transplantation Network (OPTN) website. You might also think about joining a program like the living kidney donor paired exchange or chain.

1. Please modify the text to make it easier to understand for someone who reads at or below an 8th grade level: "What is living donation? Living donation is when a living person donates an organ or part of an organ to a person in need of a transplant. Living kidney donation is possible because we can live a healthy life with one functioning kidney. Living liver donation is possible because the liver consists of two lobes, one of which can be donated to someone in need. Both lobes will regenerate to normal size and function generally within 6-12 weeks."

ChatGPT

ChatGPT

Living donation means a person who is alive gives an organ or a part of an organ to someone who needs a new one. You can give one of your kidneys to someone else because your body can work well with just one kidney. You can also give part of your liver because the liver has two parts, and you can give one away. The good news is, both parts of the liver can grow back to their normal size and work just like before in about 6 to 12 weeks.

CH

You

2. Please modify the text to make it easier to understand for someone who reads at or below an 8th grade level: "Why is living donation important? Living donation offers another choice for transplant candidates, and reduces the number of people on the national transplant waiting list (from deceased donors). Even better, kidney and liver patients who are able to receive a living donor transplant can receive a good quality organ much sooner, often in less than a year. The limited number of deceased donor organs cannot meet the growing need for lifesaving organ transplants. Living donation is one way we can give more people access to the lifesaving transplant they need. Other benefits of living donation include: The recipient can be transplanted while in better health and better able to tolerate the surgery. Living donor organs are often better quality and on average last longer than deceased donor organs. Kidney disease patients can avoid years of dialysis. The surgery can be scheduled at a time convenient for both the donor and the recipient. Many living donors refer to the experience as one of their most rewarding achievements."

ChatGPT

ChatGPT

Living donation is important because it gives people who need a transplant another option and helps reduce the long wait list for organs that come from donors who have passed away. People who need a kidney or liver can get a high-quality organ much faster, usually in less than a year, with living donation.

There aren't enough organs from donors who have passed away to meet the increasing need for life-saving transplants. Living donation lets more people get the life-saving transplant they need. Some other good things about living donation are:

- The person getting the organ can be healthier and more ready for surgery.
- Organs from living donors often work better and last longer than those from donors who have passed away.
- People with kidney disease might not have to go through years of dialysis.

- The surgery can be planned when it works best for both the person giving the organ and the one getting it.
- Many people who donate an organ feel really good about it and see it as one of the best things they've done.

CH

You

3. Please modify the text to make it easier to understand for someone who reads at or below an 8th grade level: "Does the living donor need to know the person to whom they donate an organ? No. A person may donate to a specific person they know or anonymously to someone in need. This would be a non-directed donation."

ChatGPT

ChatGPT

Does the person giving the organ need to know the person getting it? No. You can give an organ to someone you know or to someone you don't know without telling them who you are. This is called a non-directed donation.

CH

You

4. Please modify the text to make it easier to understand for someone who reads at or below an 8th grade level: "What is involved in the evaluation to be a living donor? The evaluation is designed to protect both the donor and the recipient. It ensures that the donor is healthy enough for the surgery and is making an informed decision. A potential living donor undergoes both physical and psychosocial examinations. Testing can vary depending on the organ and donor's age. In addition, routine health screens will need to be up to date before donation can occur. Learn more about living donation health screens from the American Society of Transplantation (AST)."

ChatGPT

ChatGPT

What does it take to check if you can give an organ while you're alive? The check-up is to make sure both the person giving the organ and the one getting it are safe. It checks that the person giving the organ is healthy enough for the operation and understands what they're doing. A person who wants to give an organ will have to go through health and mental health tests. What tests you need can change based on what organ you're giving and how old you are. Also, you need to make sure all your regular health checks are up to date before you can give an organ. You can learn more about the health checks for giving an organ from the American Society of Transplantation (AST).

CH

You

5. Please modify the text to make it easier to understand for someone who reads at or below an 8th grade level: "What is paired kidney donation and living donation chains? Sometimes a transplant candidate has someone who wants to donate a kidney to them, but tests reveal that the kidney would not be a good medical match. Paired donation, also called paired kidney exchange, gives that transplant candidate another option. In paired donation, two or more pairs of living kidney donors and the individuals they want to donate to are swapped to

form a compatible medical match. For more detailed information, check with your transplant hospital or learn more about paired kidney donation from UNOS.org"

ChatGPT

ChatGPT

What are paired kidney donation and living donation chains? Imagine someone needs a new kidney, and they have a friend or family member who wants to give them one. But sometimes, tests show that the friend's kidney isn't a good match for them. This is where paired kidney donation comes in. It's also called a kidney swap. Here's how it works: two or more pairs of people (one person needing a kidney and one person giving a kidney) are matched up so that everyone can get a kidney that works well for them. If you want to know more about this, you can ask your transplant hospital or look up paired kidney donation on UNOS.org.

CH

You

6. Please modify the text to make it easier to understand for someone who reads at or below an 8th grade level: "Can living kidney donors live a healthy life with a single kidney? Studies suggest that people can live a normal, healthy life with one kidney. Living kidney donors are carefully screened to ensure they are healthy before living donation can take place. They are encouraged to have regular checkups and follow a healthy lifestyle."

ChatGPT

ChatGPT

Can people who give one of their kidneys live a healthy life with just one kidney? Yes, research shows that you can live a normal and healthy life with just one kidney. Before someone can give a kidney, they go through a lot of checks to make sure they are healthy. After giving a kidney, they should go to the doctor for regular checkups and live a healthy lifestyle.

CH

You

7. Please modify the text to make it easier to understand for someone who reads at or below an 8th grade level: "Does living organ donation shorten the donor's life expectancy? No, there is no evidence that living donation shortens a lifespan."

ChatGPT

ChatGPT

Does giving an organ while you're alive make you live a shorter life? No, there's no proof that it makes you live any less time.

CH

You

8. Please modify the text to make it easier to understand for someone who reads at or below an 8th grade level: "Are living kidney donors more likely to get kidney disease? Living kidney donors are counseled about their individual risks associated with donation. The transplant team educates donors about the expected post-donation kidney function. In general, the risk of kidney disease does not exceed that of the general population with the same demographic profile."

ChatGPT

ChatGPT

Are people who give a kidney more likely to get kidney disease? People who give a kidney are told about the risks that come with giving one. The team that does the transplant talks to donors about how their kidney will work after they give one away. Usually, the chance of getting kidney disease isn't higher than it is for other people who are similar in age and health.

CH

You

9. Please modify the text to make it easier to understand for someone who reads at or below an 8th grade level: "Do living donors have to take medications for the rest of their lives? Generally, prescriptions for pain and stool softeners will be necessary in the immediate post-operative period only."

ChatGPT

ChatGPT

Do people who give an organ have to take medicine forever? No, mostly they'll just need medicine for pain and to help with going to the bathroom right after the surgery.

CH

You

10. Please modify the text to make it easier to understand for someone who reads at or below an 8th grade level: "Will a living donor be in the hospital for an extended period of time after surgery? A kidney donor will be hospitalized on average for 1 to 2 nights. A liver donor will be hospitalized on average for 4 to 7 nights. Liver donors may spend at least 1 night in the ICU for monitoring. A donor will have some follow-up appointments after hospital discharge."

ChatGPT

ChatGPT

Will someone who gives an organ stay in the hospital for a long time after the surgery? A person who gives a kidney usually stays in the hospital for about 1 to 2 nights. A person who gives part of their liver stays for about 4 to 7 nights. Someone who gives part of their liver might also spend at least one night in a special part of the hospital called the ICU to be watched closely. After leaving the hospital, they will need to go back for a few check-ups.

CH

You

11. Please modify the text to make it easier to understand for someone who reads at or below an 8th grade level: "Does a living donor have to follow a new diet plan following donation? A donor should eat a healthy, well-balanced diet, but there are no dietary restrictions."

ChatGPT

ChatGPT

Does someone who gives an organ have to eat differently after they give it? A person who gives an organ should eat healthy foods, but they don't have to follow a special diet.

CH

You

12. Please modify the text to make it easier to understand for someone who reads at or below an 8th grade level: "Can a living donor consume alcohol following donation? Excessive alcohol use is always dangerous. For liver donors, alcohol abstinence should occur for one month prior to surgery and for three months following donation. No specific restrictions for kidney donors."

ChatGPT

ChatGPT

Can someone who gave an organ drink alcohol after? Drinking too much alcohol is always bad. If someone gave part of their liver, they shouldn't drink alcohol for one month before the surgery and for three months after giving. There are no special rules about alcohol for people who gave a kidney.

CH

You

13. Please modify the text to make it easier to understand for someone who reads at or below an 8th grade level: "Should a living donor avoid pregnancy after donation? A donor should wait to become pregnant until medically cleared after donation."

ChatGPT

ChatGPT

Should someone who gave an organ wait before getting pregnant? Yes, a person should wait until a doctor says it's okay to try to get pregnant after giving an organ.

CH

You

14. Please modify the text to make it easier to understand for someone who reads at or below an 8th grade level: "Will a living donor's sex life be negatively affected by donation? Donors may engage in sexual activity when they feel well enough to do so."

ChatGPT

ChatGPT

Will giving an organ affect someone's sex life in a bad way? No, a person can have sex when they feel good enough to do it after giving an organ.

CH

You

15. Please modify the text to make it easier to understand for someone who reads at or below an 8th grade level: "Who pays for the medical expenses related to living donation? The costs for the medical testing, surgery and post-operative care are covered by the transplant hospital or recipient's health insurance. However, the living donor is responsible for the cost of routine health screens and treatment of any medical issues discovered during the evaluation. The cost of treatment of future issues that result from the donation, may be covered. Medical costs that are not covered include post-surgical routine health maintenance. Employed living donors should learn about their company's paid sick leave, disability and the Family and Medical Leave Act (FMLA) policies if applicable. A transplant financial coordinator is available at each transplant hospital to answer questions."

ChatGPT

ChatGPT

Who pays for the doctor bills when someone gives an organ? The hospital or the person getting the organ's health insurance pays for the tests, the surgery, and taking care of the donor after surgery. But, the person giving the organ has to pay for their regular health checks and any health problems found when they are checked to see if they can give an organ. If there are health problems later because of giving the organ, those might be paid for. Costs that are not covered include check-ups after surgery. People who are working should ask about their job's rules for taking paid time off, disability, and the Family and Medical Leave Act (FMLA) if it applies. Each hospital has someone who can answer questions about paying for these things.

CH

You

16. Please modify the text to make it easier to understand for someone who reads at or below an 8th grade level: "Will the living donor have any out of pocket expenses? Yes. It varies case by case and could include lost wages, travel expenses, childcare costs, etc. The National Organ Transplant Act allows for organ recipients to pay their living donors' travel and housing in connection with the donation. The transplant hospital financial coordinator will check with the insurance company of the intended recipient as the policy may provide a travel benefit. There are organizations that provide financial resources to living donors for uncovered costs depending on need. Transplant hospitals can provide additional information about options. National Living Donor Assistance Center (NLDAC) Note that eligibility for financial assistance from NLDAC is based on the recipient's household income which may not be more than 300% of the current Federal Poverty Guidelines."

ChatGPT

ChatGPT

Will the person giving the organ have to pay for anything themselves? Yes, it can change depending on the situation. They might need to pay for things like missing work, traveling, and taking care of kids. The law says that the person getting the organ can help pay for the donor's travel and place to stay. The hospital's financial person will talk to the insurance company to see if they can help with travel costs. There are groups that help donors with money for things insurance doesn't cover, if they really need it. Hospitals can tell you more about these options.

There's also a special place called the National Living Donor Assistance Center (NLDAC) that helps, but to get help, the income of the person getting the organ can't be more than three times the poverty level.

CH

You

17. Please modify the text to make it easier to understand for someone who reads at or below an 8th grade level: "Will a living donor have trouble getting health insurance or life insurance after they donate? Being a living donor may affect the ability to get health or life insurance. The transplant team can provide details."

ChatGPT

ChatGPT

Could giving an organ make it hard to get health or life insurance later? Yes, it might. The team that does the transplant can tell you more about this.

CH

You

18. Please modify the text to make it easier to understand for someone who reads at or below an 8th grade level: "How risky is living donor surgery? The surgery to donate a kidney is generally safe, with a 3 in 10,000 risk of death. The risks for living liver donation surgery are slightly higher, dependent on the amount of liver donated. More information about living donation risks is available on the United Network for Organ Sharing (UNOS) website, TransplantLiving.org. As with any surgery, living-liver donation can include complications such as bleeding, infection, bile duct problems, and blood clots, etc. Complications occur in about 16% of cases. Living donors are carefully evaluated prior to a living-donor liver transplant to ensure that risk is minimized. The estimated mortality is about 0.5% (5/1000), depending on which lobe (part) of the liver is donated."

ChatGPT

ChatGPT

How dangerous is the surgery for giving an organ while you're alive? Giving a kidney is mostly safe. The chance of dying is very small, like 3 out of 10,000 people. Giving part of your liver is a bit riskier, and how risky it is depends on how much liver you give. You can find out more about the risks on the United Network for Organ Sharing (UNOS) website, TransplantLiving.org. Like any surgery, giving part of your liver can have problems like bleeding, getting an infection, issues with the bile duct, and blood clots. About 16 out of 100 people have these problems. Doctors make sure to check donors carefully before they give part of their liver to keep risks low. The chance of dying is about 0.5% (5 out of 1000 people), and it depends on which part of the liver is given.

CH

You

19. Please modify the text to make it easier to understand for someone who reads at or below an 8th grade level: "Can adults over age 50 donate? Yes, donors must be healthy and can donate primarily on a basis of health. Some transplant hospitals may have age restrictions."

ChatGPT

ChatGPT

Can people older than 50 give an organ? Yes, if they are healthy, they can give an organ. It really depends on how healthy they are. But, some hospitals might have rules about how old you can be to give an organ.

CH

You

20. Please modify the text to make it easier to understand for someone who reads at or below an 8th grade level: "Can members of the LGBTQ+ community be living donors? There are no restrictions based on a potential donor’s LGBTQ+ identification. All potential living donors undergo a thorough medical evaluation to determine if donation is safe."

ChatGPT

ChatGPT

Can LGBTQ+ people give an organ while they're alive? Yes, being LGBTQ+ doesn't stop someone from giving an organ. Everyone who wants to give an organ goes through a big health check to make sure it's safe for them to do so.

CH

You

21. Please modify the text to make it easier to understand for someone who reads at or below an 8th grade level: "Can people living with HIV be living donors? The HIV Organ Policy Equity Act (HOPE) Act provides donation and transplantation opportunities for people living with HIV with minimal additional testing at specific centers. Refer to the OPTN HOPE (HIV Organ Policy Equity) Act page for more information."

ChatGPT

ChatGPT

Can people with HIV give an organ while they're alive? Yes, there's a special law called the HOPE Act that lets people with HIV give and receive organs. They need a little extra testing at certain places. You can look up the OPTN HOPE Act page to learn more.

CH

You

22. Please modify the text to make it easier to understand for someone who reads at or below an 8th grade level: "Are people with tattoos able to be living donors? All potential living donors undergo a thorough medical evaluation to determine if donation is safe for both the donor and recipient."

ChatGPT

ChatGPT

Can people with tattoos give an organ while they're alive? Yes, everyone who wants to give an organ is checked carefully by doctors to make sure it's safe for them and the person getting the organ. Having tattoos doesn't automatically stop someone from being able to donate.

CH

You

23. Please modify the text to make it easier to understand for someone who reads at or below an 8th grade level: "What happens if a living donor changes their mind? Being a living donor is completely voluntary. The donor can delay or stop the process at any time. Their reasons for choosing to not donate will remain confidential and will not be shared with the recipient or anyone else."

ChatGPT

ChatGPT

What if someone who wants to give an organ changes their mind? Giving an organ is a choice. If someone decides they don't want to give an organ anymore, they can stop or wait at any time. The reasons why they don't want to give an organ will be kept secret and not told to the person who was going to get the organ or anyone else.

CH

You

24. Please modify the text to make it easier to understand for someone who reads at or below an 8th grade level: "Do living donors ever share their experiences and what are some resources? Yes, 97% of living donors say they would donate again and are excited to share their experiences. Donate Life WELD (We Encourage Living Donation), a program of Donate Life

America, provides a forum for living donors to share their stories with members of their community. To learn more about DL/WELD, contact DL/WELD coordinator. View stories from living donors and recipients."

ChatGPT

ChatGPT

Do people who have given an organ talk about what it was like? Yes, almost all of them, 97% of people who have given an organ, say they would do it again and they like to tell others about their experience. There's a special group called Donate Life WELD (We Encourage Living Donation) that helps people who have given an organ share their stories with other people. If you want to know more about this group, you can get in touch with the person who organizes DL/WELD. They also have stories from people who have given and received organs that you can read.

CH

You

25. Please modify the text to make it easier to understand for someone who reads at or below an 8th grade level: "How would a potential living donor start the process if they want to donate to a specific person? Contacting the transplant hospital of the person in need of the transplant is the first step. Many hospitals have a form on their website potential donors can submit to begin the process or they can call the transplant hospital and ask to speak with the living donor coordinator for the organ they wish to donate."

ChatGPT

ChatGPT

If someone wants to give an organ to a certain person, how do they start? The first thing to do is get in touch with the hospital where the person needing the organ is being treated. A lot of hospitals have a form on their website that you can fill out to start the process, or you can call the hospital and ask to talk to the person who organizes living donations for the organ you want to give.

CH

You

26. Please modify the text to make it easier to understand for someone who reads at or below an 8th grade level: "What if the intended living kidney donor and intended recipient are not a match? Begin by talking to the transplant hospital where the intended recipient is listed to see if they participate in paired donation. The OPTN Kidney Paired Donation Program facilitates paired donation between transplant hospitals."

ChatGPT

ChatGPT

What if the person who wants to give a kidney and the person who needs it don't match? First, you should talk to the hospital where the person who needs the kidney is going to get their transplant to see if they do kidney swaps, also known as paired donation. The OPTN Kidney Paired Donation Program helps set up these swaps between hospitals.

CH

You

27. Please modify the text to make it easier to understand for someone who reads at or below an 8th grade level: "What is the first step for a potential living donor who does not have a specific recipient in mind? Please refer to the Organ Procurement and Transplantation

Network (OPTN) member list of all U.S. transplant hospitals. You may also want to consider being part of a living kidney donor paired exchange or chain."

ChatGPT

ChatGPT

What should someone do first if they want to give an organ but don't have someone specific they want to give it to? You can look at the list of transplant hospitals in the U.S. on the Organ Procurement and Transplantation Network (OPTN) website. Another option is to think about joining a kidney swap or chain, where your kidney goes to someone you match with, and someone else gets a kidney that matches them.
